# Editorial: Endocrine-active substances in food: identification, evaluation, and control

**DOI:** 10.3389/fnut.2023.1252122

**Published:** 2023-08-11

**Authors:** Yu-Syuan Luo, Xiaoqing Ye, Shanshan Yin

**Affiliations:** ^1^College of Public Health, Institute of Food Safety and Health, National Taiwan University, Taipei, Taiwan; ^2^Master of Public Health Degree Program, College of Public Health, National Taiwan University, Taipei, Taiwan; ^3^School of Medical Technology and Information Engineering, Zhejiang Chinese Medical University, Hangzhou, China; ^4^Key Laboratory of Pollution Exposure and Health Intervention of Zhejiang Province, Interdisciplinary Research Academy (IRA), Zhejiang Shuren University, Hangzhou, China; ^5^Toxicological Centre, University of Antwerp, Wilrijk, Belgium

**Keywords:** endocrine disruptors, diet, metabolic disease, estrogenic activities, ultra-processed food

Diet is crucial in maintaining life, yet harmful substances can enter the human body via dietary exposure. Humans can be exposed to harmful substances indirectly from food contact materials [e.g., di(2-ethylhexyl)phthalate, DEHP] or directly from food [e.g., pesticides (1, 2), soybean, and ultra-processed food]. Chronic exposure to these substances may lead to the development of chronic diseases; endocrine disruption is an early molecular event that plays an essential role in disease progression (3). Endocrine disruptors are exogenous chemicals that interfere with endocrine functions via direct binding with nuclear receptors, such as estrogen and androgen receptors (4). Studies on endocrine-disrupting chemicals in food have received increasing attention (5–7); however, our understanding of endocrine-disrupting potentials of foodborne chemicals, the interplay among endocrine-active substances, gut microbiota, and metabolic syndromes, and how to mitigate the adverse effects caused by endocrine disruptors are still limited.

This Research Topic aims to better characterize the exposure, health effects, and mitigation of endocrine-active substances in food. This special e-collection includes four papers studying the relationships between endocrine disruptor exposure and health effects (Lin et al.; Shu et al.) to develop a novel tool for the rapid screening of estrogenic potential (Debon et al.) and investigate a potential mitigation strategy using Mexican food supplementation (Rosas-Campos et al.).

Using data from 13,634 participants in the National Health and Nutrition Examination Survey (NHANES), Lin et al. sought to examine the relationships among daily dietary fiber intake (DDIF), short sleep duration (SSD), and DEHP exposure. The authors found that higher DEHP exposure was associated with an increased risk of developing SSD. Interestingly, the association between DEHP exposure and SSD was sex-specific, with a significant interaction observed. In addition, for SSD <7 h, a significant interaction was observed between DEHP and DDIF tertiles. In conclusion, this study showed an adverse association between DEHP exposure and short sleep duration. However, it also identified a potential ameliorating effect of median DDIF on short sleep duration in the presence of DEHP exposure. These findings suggest that the intake of dietary fiber may play a protective role against the DEHP-induced adverse effects on sleep duration.

A systematic review and meta-analysis on the association between ultra-processed food (UPF) consumption and the risk of metabolic syndrome was conducted by Shu et al.. The analysis included nine studies, involving 23,500 participants and 6,192 cases with metabolic syndrome. Pooled analysis showed a positive association between the highest UPF category and the risk of developing metabolic syndrome, while the stratified analyses showed that this association was significant in cross-sectional studies but not in cohort studies. Additionally, the association between UPF consumption and increased risk of metabolic syndrome was more pronounced in studies with lower quality scores (<7) compared to studies with higher quality scores (≥7). Significant associations were also found when analyzing studies based on sample size. These findings suggest that a higher level of UPF consumption is significantly associated with an increased risk of metabolic syndrome, but further longitudinal studies are needed to confirm this association.

Current analysis of endocrine disruptors in food is primarily based on prior knowledge, which may neglect compounds with unknown endocrine-disrupting potential. To overcome this limitation, Debon et al. used a promising approach that combines high-performance thin-layer chromatography (HPTLC) with bioassays to rapidly determine the estrogenic activity of test compounds. Seven isoflavones were identified in the soy isolates; the results showed correlations between the extracts of the soy isolates and the identified isoflavones. The p-YES assay identified an estrogenic bioactive zone. Bioactive zone analysis further highlighted signals corresponding to the previously identified isoflavones and two additional isoflavones. This study established a dose-response relationship for estrogenic activity in both bioassays for all detected isoflavones, while the most active compounds were identified as genistein, daidzein, and naringenin. A concordance analysis integrating the analytical and bioassay data indicated that genistein and daidzein were the primary drivers of estrogenic activity in the soy protein isolates. The authors adopted an integrated approach for identifying the bioactive component (i.e., estrogenic) in soy extracts, illustrating a powerful workflow for characterizing the endocrine activity of complex mixtures.

Lastly, the researchers from Mexico (Rosas-Campos et al.) investigated the effects of a supplementation mixture of Mexican functional foods (MexMix) consisting of Opuntia ficus indica (nopal), Theobroma cacao, and edible crickets on an obesogenic mice model compared to a high-fat and fructose/sucrose diet. The results showed that the MexMix supplementation led to reduced body weight, liver weight, visceral fat, and epididymal fat compared to the high-fat diet group. Additionally, MexMix supplementation resulted in decreased levels of triglycerides, cholesterol, LDL cholesterol, insulin, glucose, GIP, leptin, PAI-1, and resistin. Analysis of the gut microbiota revealed that MexMix supplementation increased the abundance of beneficial bacteria such as Lachnospira, Eubacterium coprostanoligenes, and Blautia. These findings suggest that MexMix food supplementation may serve as a potential strategy for the treatment of obesity and related diseases associated with the excessive intake of fats and sugars.

The articles in this e-collection provide important insights into dietary exposures, health effects, and potential interventions for endocrine disruptor-induced effects. Given that only four articles were retained in this e-collection after detailed curation, more studies in this field are warranted to fully understand emerging endocrine disruptors and their potential influence on human health. The papers published in this e-collection share the practical efforts of the research on endocrine disruptors in food. We hope that this e-collection would inspire more studies on endocrine disruptors in food, deciphering the intriguing interplay among environmental chemicals, diets, and diseases.

## Author contributions

Y-SL wrote the introduction and conclusion. SY wrote the central part with comments on the cited papers and references. XY reviewed and edited the original draft. All authors contributed to the article and approved the submitted version.
